# Hybridizing video-based learning with simulation for flipping the clinical skills learning at a university hospital in Pakistan

**DOI:** 10.1186/s12909-023-04580-y

**Published:** 2023-08-21

**Authors:** Sana Saeed, Maryam Hameed Khan, Muhammad Muneeb Ullah Siddiqui, Anny Dhanwani, Areeba Hussain, Muhammad Maisam Ali

**Affiliations:** 1 Department of Paediatrics and Child Health, Aga Khan University, Karachi, Pakistan; 2 Department of Community Health Sciences, Resident Physician, Aga Khan University, Karachi, Pakistan; 3 Medical Student, Aga Khan University, Karachi, Pakistan

**Keywords:** Flipped classroom, Clinical teaching, Clinical skills, Video-based learning

## Abstract

**Background:**

While learning and practicing on actual patients is a major mode of teaching clinical skills, concerns about patient safety, unavailability, and lack of standardization have led to the development of simulation for medical education. Simulation-based teaching is affected by challenges such as lack of protected time for faculty, inexperienced learners, and the number of students per group. These have led to the integration of various eLearning formats in the curriculum. The hybridized format changes the traditional clinical skills teaching into the flipped classroom. This study aims to measure the effectiveness of hybridizing video-based learning with simulation for flipping the clinical skills teaching of fourth-year medical students at the Department of Paediatrics and Child Health at Aga Khan University, Pakistan.

**Methods:**

The study employed a mixed-methods design. Fourth-year medical students of the batch 2020-21 (n = 100) consented to participate in the study. The quantitative component focuses on identifying the effect of the intervention on the perceived self-efficacy of medical students (batch 2020-21) relevant to the clinical skill. Along with this, the performance of the intervention batch of 2020-21 on the end of clerkship objective structured clinical exam (OSCE) was compared with the previous batch of 2019-20, taught using simulation alone. Focused group discussions (FGDs) were used to explore the experiences of medical students (batch 2020-21) about the intervention. Quantitative data underwent descriptive and inferential analysis using Stata v16 while qualitative data underwent content analysis using NVivo software.

**Results:**

Hybridization of video-based learning with simulation significantly improved self-efficacy scores for all examinations (cardiovascular, respiratory, neurological, and abdomen) with p-value < 0.05. OSCE scores of the intervention group were significantly higher on the neurological and abdominal stations as compared to the previous batch (p-value < 0.05). In addition, the overall structure of the intervention was appreciated by all the students, who stated it allowed reinforcement of basic concepts, retention, and further insight into clinical applications.

**Conclusion:**

The hybridization of video-based learning with simulation facilitated in creation of better opportunities for medical students to revive their prior knowledge, apply core concepts for the problem and engage in clinical reasoning.

## Background

Experiential learning, or ‘‘learning by doing’’, [1] has long dominated the culture of professional apprenticeships. In medical training, however, this powerful pedagogical technique carries an inherent risk to patients. ‘‘See one, do one, teach one’’ has been a guiding principle of medical trainees, yet leaves precious little room for error [2]. Clinical skills are an essential component of medical education, reflecting the practitioners’ medical knowledge, assessment, and analytical skills that help lead to a diagnosis and ultimately impact patient outcomes. While learning and practicing on actual patients is a major mode of teaching clinical skills, [3] concerns about patient safety, unavailability of patients for learning, and lack of standardization have led to the introduction of simulation in medical education [4]. Employing medical simulation techniques can help in shifting medical training from the old “See one, do one, teach one” method into a “see one, practice many, and do one” model of success. They also cultivate a safe learning environment, enhance teamwork and student satisfaction, and optimize learning [5, 6]. The learners are permitted to err and are provided with the chance to practice and to receive constructive feedback which, it is hoped, will prevent making the repetition of such mistakes in real patients less likely [7].

Providing learners with opportunities for deliberate practice is of utmost importance during simulation-based experiences. The performance should be closely observed by the instructor and appropriately debriefed for effective learning. It helps in optimizing the learning curve and facilitates serving the individual needs of learners. Lack of protected time for faculty, inexperienced learners, and the number of students in groups affect the opportunities for deliberate practice during simulation-based teaching. Similarly, on many occasions, most of the in-class time is invested in establishing those concepts, leaving less room for application for students, which is essential for deeper learning. These challenges led to integrating various eLearning formats in the medical curriculum. The use of online videos for the purpose of teaching clinical skills is reported in literature both in isolated and hybridized formats [8, 9]. The hybridized format changes the traditional clinical skills teaching into the flipped classroom. The flipped classroom (FC) is an inverted pedagogical approach where learners learn the basic content independently before class, such as by watching a learning video on the online platform [10]. The in-class time is invested in application-based learning that is shown to improve the performance on higher cognitive tasks, promote order problem solving and enhance clinical reasoning skills [11–13]. Various studies have demonstrated the effectiveness of using blended learning, flipped classrooms, and simulation-based teaching in the local context of low-middle-income countries [14–16]. A study from Pakistan demonstrated the use of hybrid learning in teaching anatomy to dental students. This study reported significantly improved performance of students as compared to conventional teaching. It also showed enhanced satisfaction of the students with the hybrid pedagogical approach [17]. Another study reported the effectiveness of using the online modality for teaching clinical skills to pre-clinical students in Pakistan. This study also showed significantly improved performance and satisfaction of participants with the process of learning [18]. However, there is a dearth of literature demonstrating the utilization of flipped classrooms and simulation in the hybrid form for teaching clinical skills in the undergraduate and postgraduate systems of education in Pakistan.

The MBBS Year IV paediatric clerkship at Aga Khan University is an 8-week rotation in which undergraduate students rotate through general paediatrics, neonatology, cardiology, and neurology. Previously, the teaching strategies utilized were bedside teaching, clinics, large class formats, and small group learning (e.g., tutorials). The students are assessed through the end of clerkship Objective Structured Clinical Exams (OSCE) and end of professional, theory exam (multiple choice questions and short answer questions). In 2018-19, simulation-based teaching was integrated into the curriculum for teaching paediatric examination skills.

These sessions, although helped to learn the systematic approach to common paediatric clinical examinations, lacked the optimum opportunity for the application of those skills to understand the pathophysiology in each situation, thereby affecting the process of clinical reasoning. At the end of clerkship feedback, the medical students appreciated the simulation-based skills session but suggested providing an opportunity to apply the skills and knowledge related to a given clinical examination, e.g., respiratory examination to reach a diagnosis of asthma. This study aims to measure the effectiveness of hybridizing video-based learning with simulation for the teaching of clinical skills on the perceived self-efficacy of students, the student’s satisfaction with the learning pedagogy, and their performance in their clinical exams (OSCE).

## Materials and methods

### Study design

We used a mixed-method study design. The quantitative arm was based on a quasi-experimental approach which was meant to ascertain the effectiveness of the revised pedagogical framework on the clinical skills learning of students. To explore the experiences of medical students, we conducted focused group discussions. Ethical approval was obtained through the Ethical Review Committee of The Aga Khan University (2020-5560-15128).

### Setting and participants

This study was conducted at the Department of Paediatrics and Child Health at Aga Khan University (AKU), Karachi, Pakistan. AKU offers a five-year Bachelor of Medicine, Bachelor of Surgery (MBBS) program which constitutes the initial two years as pre-clinical years followed by three clinical (clerkship) years.

**Fig. 1:**
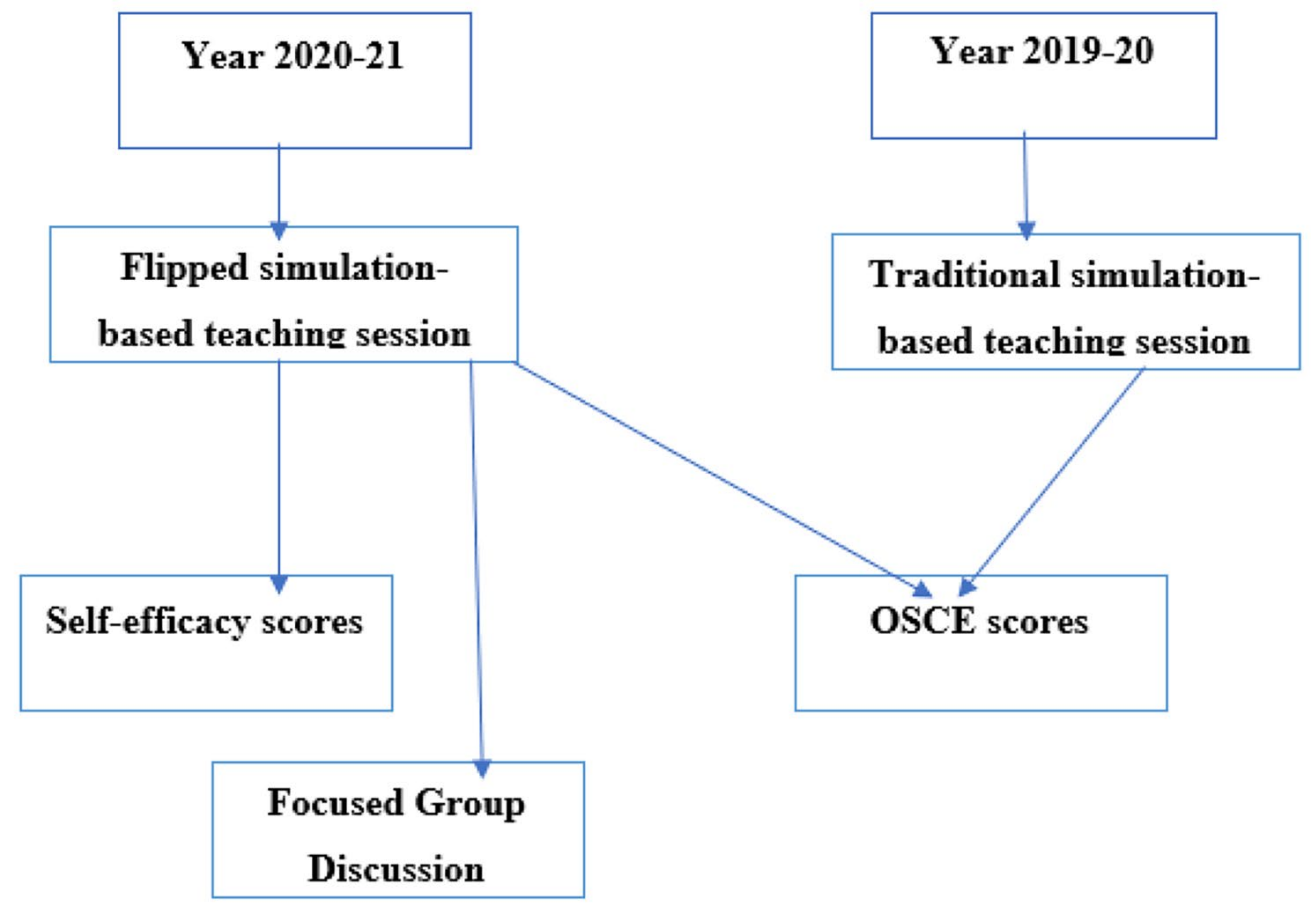
Flow diagram of methodology scheme

Students of the academic batch 2020-21 (n = 100), who attended all the flipped-style clinical skills and provided consent were included in this study through convenient sampling. Students were informed of this study, and all students of the batch of 2020-21 provided written consent to participate. All the medical students signed the consent. After signing the consent MBBS batch of 2020-21 participated in quantitative and qualitative arm of data collection. For the batch of 2019-20, as there was no human-to-human contact for the purpose of data collection and the data obtained was anonymous without any personal identifier, obtaining consent was exempted. Due to the integration of the intervention within the curriculum, it was not possible to identify the control within the same group, hence students of the previous batch (2019-20), who were taught using traditional simulation-based teaching, were used for comparison (n = 100) (Fig. 1). This made the total sample of two to address one of the study objectives i.e., to evaluate the effectiveness of using flipped-style simulation-based teaching on the clinical skills of medical students. This study was conducted from February 2021 to January 2022.

### The framework of clinical teaching sessions

The paediatric clerkship curriculum was reviewed by a team of clinicians and medical educationists. Two essential clinical cases were identified as relevant to each clinical presentation (e.g., asthma and ventricular septal defect for a child with respiratory distress), and one clinical presentation was developed for each paediatric examination skills, e.g., “an 8-year-old boy is admitted in paediatric ward with abdominal pain and fever”. Each session had a pre-class learning component which was aimed to refresh the prior knowledge regarding relevant skills. The videos were developed by the project team with the faculty members who demonstrated the relevant skill on a standardized patient. Each video had 2–3 quiz questions which were embedded within the videos. These questions were relevant to the clinical examination in the video. Video links were shared with the students three days prior to the session. The students were informed about the overall session plan in an orientation session. The expectation regarding the pre-class learning was shared in the session. The program administrator recorded and shared the names of the students who access the link and attempted the questions while watching the videos. Each session was of an hour and a half in duration and had 8–10 students. The students worked in groups to understand, demonstrate, and illicit relevant clinical skills on the given medium fidelity mannequin (Mega Kid Code from Laerdal) to reach a diagnosis. On each step, they were debriefed by the facilitator (who was also a member of the project team). The students were given the opportunity to reflect upon their learning and assimilate it into their subsequent performances. This was followed by the opportunity of deliberate practice where the students practiced the skill repetitively. During this phase, focused feedback was shared to the student by the facilitator and peers to improve their performance on the given skill.

### Data collection

#### Quantitative data

The self-efficacy questionnaire was developed and validated in the pilot group. The questionnaire comprised items related to examination skills and analytical reasoning pertinent to each simulation-based scenario. The performance was rated on a 5-point scale (0 = no idea/not confident at all, and 5 = extremely confident). The questionnaire was developed by a team consisting of two medical educationists and two paediatric faculty members. This questionnaire was piloted on ten interns and the ambiguities were adjusted according to the feedback received from interns before final implementation. It was then administered pre-post skills-based sessions on the intervention batch of 2020-21. The key purpose was to see the effect of watching the video and having a simulation-based experience on students’ perceived confidence in analyzing the patient’s problem.

OSCE scores of the students on the examination skills station of the current batch (2021, taught using a combination of video-based learning and simulation) and the previous batch (2019, taught using simulation-based teaching alone) were also collected to see the effect of the intervention on students’ performance in the OSCE exam. The OSCE for the cohort of the year 2019-20 happened at the end of the clerkship (before the intervention). Anonymized scores were obtained retrospectively for comparison with the intervention group. The OSCE for the intervention group (2020-21) happened after the intervention at the end of the clerkship. The OSCE stations were not designed by the research team. This exam is part of the routine end-of-clerkship summative assessment of medical students. In summary, the pre-session videos supported refreshing the prior knowledge of the students relevant to one skill (e.g., abdominal exam). During the simulation session, the students applied that knowledge in the given scenario to reach to certain diagnosis (e.g., a child with abdominal pain), while the OSCE tested the approach in another clinical context relevant to that skill (e.g., a child with abdominal pain and jaundice).

#### Qualitative data

A semi-structured interview guide was developed in English with predetermined open-ended questions, following a relevant literature review on electronic learning and simulation and discussion within the research team. The team was comprised of two medical educationists with relevant experience in simulation-based research and two simulation expert faculty members. The focus of the FGD was to explore the experiences of medical students exposed to electronic learning and simulation. They were encouraged to share the variable challenges they faced and the possible benefits they perceived. Five focused group discussions were conducted with thirty-three medical students. Four focused groups had six students, two had five, and one group had four students respectively. This number made-up 33% of the whole batch of 2020-21. The students shared their experiences with a trained researcher with experience in qualitative research. The FGD guide consists of open-ended questions with relevant probes which were directed toward exploring the experience of students about learning through the hybrid model. There were no refusals or dropouts, and no repeat interviews were conducted. The total duration of each FGD was approximately 40–50 min and audio recordings were made. Interviews were conducted in English and were held in as much privacy as possible, with no other person apart from the participants and research investigator present during the consultations. The data was collected via face-to-face and online sessions. Reflective diaries were maintained by the investigator and all audio-recorded FGDs were transcribed verbatim in English on the same day. Transcripts were rechecked to identify discrepancies.

### Data analysis

#### Quantitative data

Data underwent descriptive statistical analysis using Stata v16. Results are reported as mean and standard deviation, and confidence intervals (CI) are also reported. Pretest and post-test self-efficacy scores were compared using paired t-tests. OSCE scores of cardiovascular examination, abdominal examination, respiratory examination, and motor examination were compared to the post-test self-efficacy scores of the same examination skills using an independent sample t-test. OSCE scores for years 2019-20 (pre-intervention) were also compared with the year 2020-21 (post-intervention) groups using an independent sample t-test. A p-value of < 0.05 was considered significant.

#### Qualitative data

Data was transcribed within 48 h of each interview. Thematic analysis was conducted using NVivo software (version 10) to manage and code interviews. The thematic approach developed by Braun and Clarke [19] was used, which included the seven steps of transcription, reading and familiarization, coding, searching for themes, reviewing themes, defining, and naming themes and finalizing the analysis. During the data analysis process, each transcript was re-read, annotated where relevant information was found, and analyzed separately by two independent researchers. Inductive coding was performed on the first FGD to develop a coding scheme, which was subsequently reviewed, discussed, and finalized, followed by coding of the remaining transcripts. Responses were coded to relevant nodes, which were later categorized into a hierarchy of tree-nodes. Once coding was completed, emerging themes and subthemes were drawn from the tree nodes which would help to answer our research objectives. The process was completed with each transcript being reviewed to ensure that all necessary information had been captured and appropriately categorized and interpreted. Verifying the accuracy of emergent codes, themes, and interpretations was achieved by constant rereading, comparing, and redefining of themes both alone and between the two researchers to ensure reliability. Discussion of interpretations was undertaken with a researcher not directly involved with the study. Direct quotations from study participants were extracted from the FGDs and included to relay critical findings.

## Results

### Quantitative data

Hundred medical students (Batch of 202) participated in the quantitative arm out of which 56% were male. Mean self-reported efficacy scores along with CI are presented in Table 1. This table also depicts the comparison of self-efficacy sores scores with paired t-tests for the intervention batch of 2020–2021. As evident from the results there is a significant difference between the students’ self-reported efficacy scores in all systems (Cardiovascular, Respiratory, Abdomen, and Motor) before and after flipping the simulation-based teaching sessions (P-value < 0.05).

**Fig. 2:**
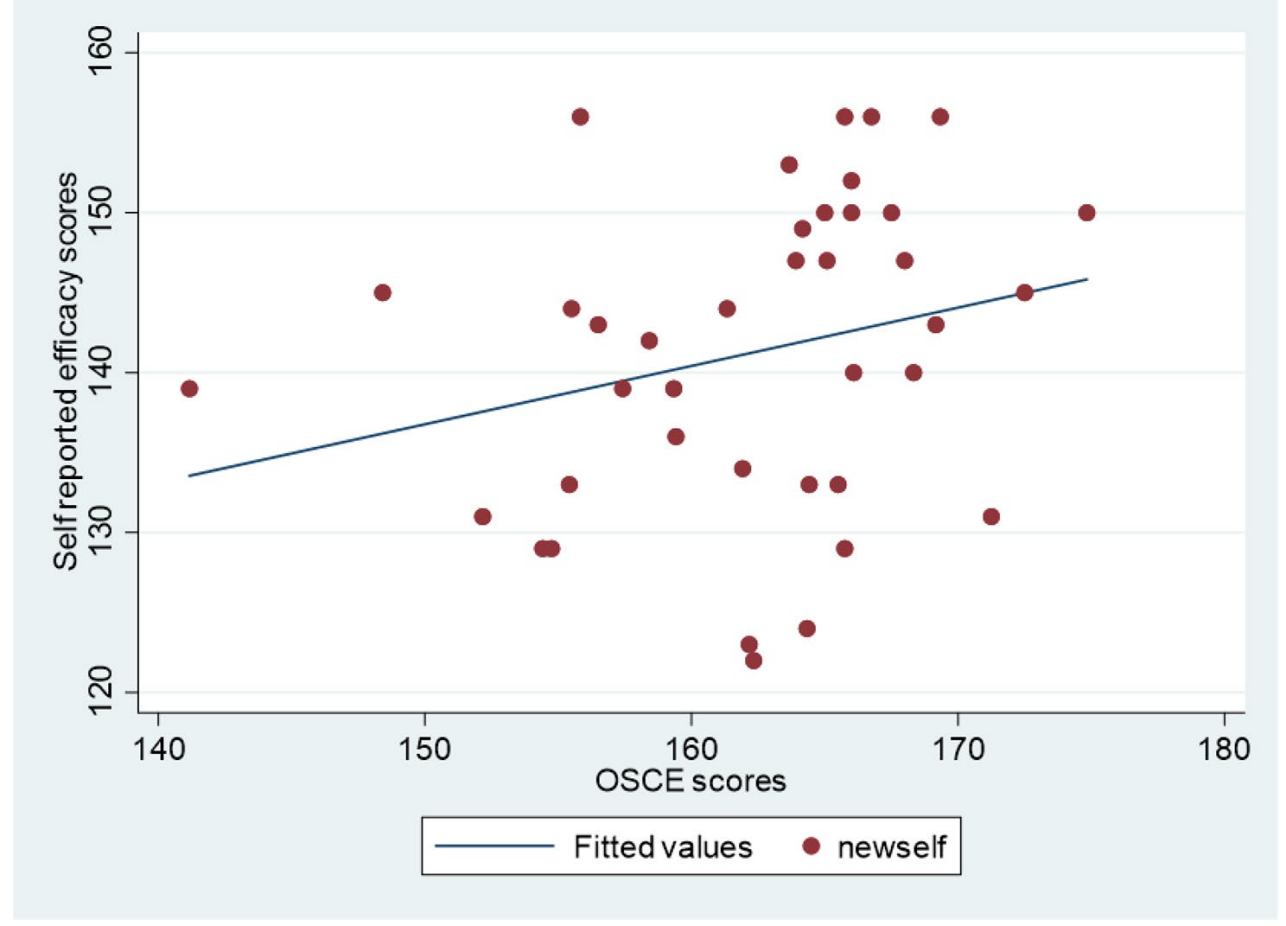
Correlation of self-reported efficacy scores and OSCE scores of the intervention group (the year 2020–2021)

The comparison of the performance in OSCE between the pre-intervention batch (2019-20), taught using simulation alone with the (batch of 2019-20) and intervention (batch of 2020-21) is shown in Table 2. There was a highly significant difference between scores of students in the abdomen and the central nervous system exams (CNS) before and after the intervention. This study reports a weak correlation (r = 0.25) between the self-reported efficacy scores and OSCE scores (Fig. 2). Possible reasons for this might be the small sample size and variability in examination skills stations which were selected in the OSCE exam.

**Table 1:** Mean self-reported efficacy scores and paired t-test statistics of students before and after the flipped-style simulation-based session

n	System	Mean ± SD	CI	T statistics(df)	P-value
82	Pretest CVS Posttest CVS	30.5 ± 4.83 40.2 ± 4.49	29.43–31.56 39.24–41.21	20.78 (81)	< 0.0001**
82					
95	Pretest Respiratory Posttest Respiratory	32.38 ± 6.32 40.35 ± 4.75	31.10–33.67 39.38–41.32	10.31 (94)	< 0.0001**
95					
92	Pretest Abdomen Posttest Abdomen	31.15 ± 5.57 40.30 ± 4.80	29.99–32.30 39.30–41.29	14.49 (91)	< 0.0001**
92					
92	Pretest CNS Posttest CNS	30.20 ± 4.56 40.30 ± 4.70	29.26–31.15 39.33–41.27	18.70 (91)	< 0.0001**
92					

*p-value < 0.05

**Table 2:** Difference between Pre-intervention (Year 2019–2020) and Post Intervention (Year 2020–2021) OSCE scores

N	System	Means Score ± SD	95%, CI	T statistics	P-value
43 98	Pretest CVS Post CVS	75.1 ± 15.29 71.8 ± 20.86	68.34–72.38 66.80–71.75	-0.936	0.82
83 57	Pretest Respiratory Post Respiratory	70.36 ± 9.26 69.07 ± 10.92	68.33–72.38 66.80–72.59	-0.37	0.64
83 39	Pretest Abdomen Post Abdomen	66.92 ± 7.29 83.89 ± 5.59	65.34–68.52 82.08–85.71	12.85	<0.001**
62 80	Pretest CNS Post CNS	74 ± 9.23 77.9 ± 9.77	71.65–76.35 75.68–80.03	2.39	0.009**

*p-value < 0.05

### Qualitative data

#### General information

**Fig. 3:**
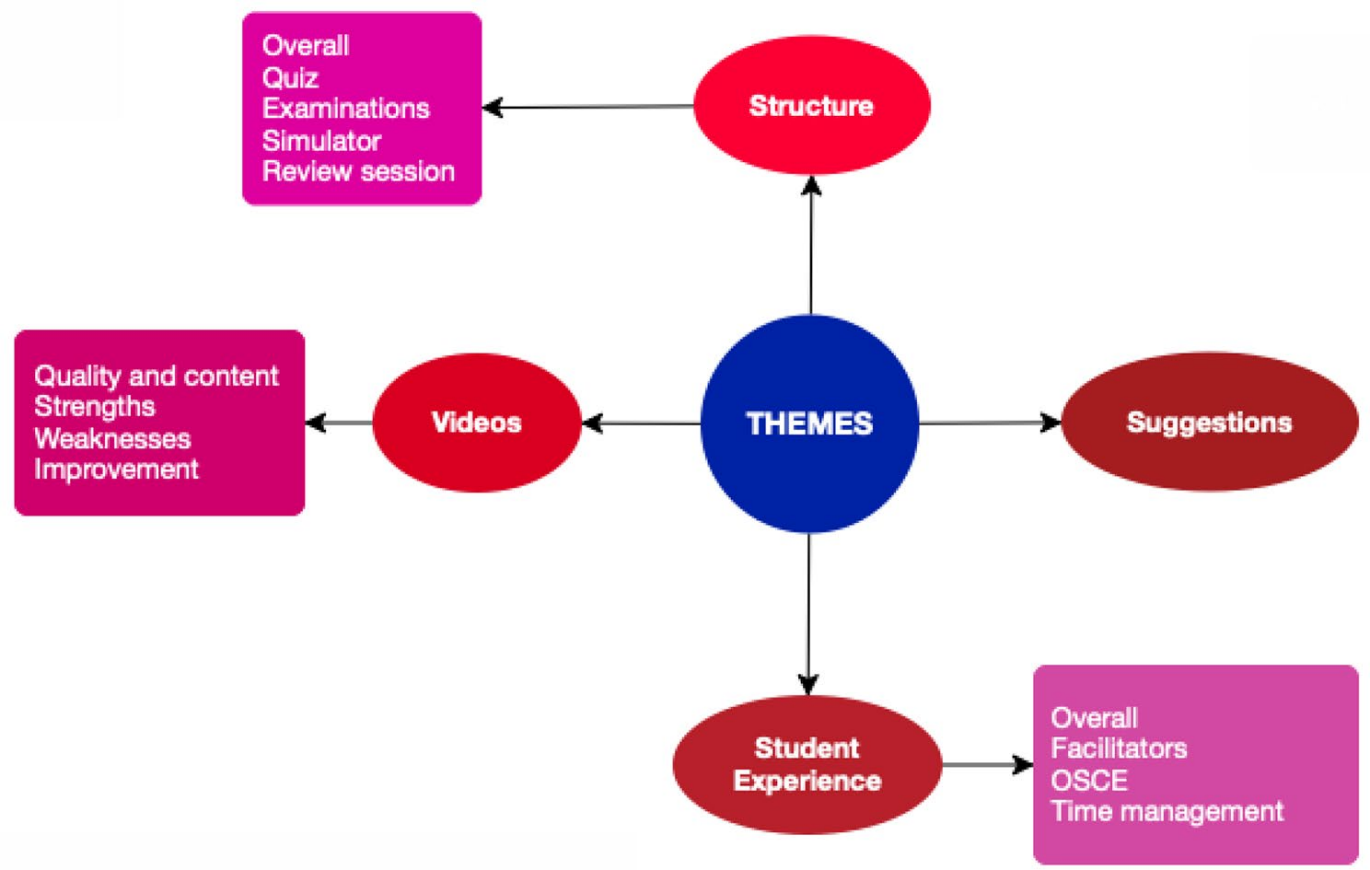
Extracted themes

The discussions explored the experiences of medical students who were exposed to electronic learning and stimulation for the teaching of examination skills, to measure the perceived self-efficacy of students in each paediatric clinical skill, the student’s satisfaction with the learning pedagogy, and their performance in clinical exams. Figure 3 displays the various themes discussed during the group discussions regarding simulation-based teaching with electronic learning.

### Structure

The overall structure of the intervention was appreciated by all the students. Initiating the experience with the educational video, followed by a quiz helped to understand and review concepts, and formulate questions that may be asked during the in-person session with a facilitator. It was helpful to learn and have knowledge beforehand so all the students in the group were on the same page and there was maximum productivity and participation during the session.

*“The videos followed by the sessions was a good idea because the videos would give us an overview and the sessions would provide extensive knowledge.” – (FGD_3_Participant 4)*

*“That revision video just before the session helped reinforce everything that we have learned so far, and then coming to the session and putting those skills to test was really helpful.” – (FGD_1_Participant 1)*

Although most of the information in the videos was known to students, it reviewed important concepts which were further reinforced during the session. Students were able to extensively build on basic concepts and gain detailed insight into the practical application of clinical skills as compared to if they attended the session without the preceding video. The final review session which comprised of all the various examinations taught gave the students another chance to practice, learn from previous mistakes and reinforce concepts. There did exist a minority view of redundancy, however, the repetition was identified as being helpful in retaining information and performing well in OSCEs.

*“About the redundant part, I feel like the more times that was repeated the more my concepts were evaluated. When I went to the OSCE, I didn’t revise my examinations very well, but I knew everything that I have to do. It was repeated so much that it was drilled into my head.” – (FGD_2_Participant 6)*

### Videos

The quality and content of the videos in the intervention should be a prime focus of improvement in future offerings (Table 3). There was unanimous agreement on the quality of needed work, with special emphasis on improving the camera angle for better viewing, having multiple cameras to show different views such as from the foot end as well as the bedside, and cameras zooming in to focus on the specific area being examined so it is easier to observe and learn the technique being shown.

Watching videos before the practical clinical skills session was beneficial; it prepared students for the session which followed by giving them baseline knowledge, and an idea of how to perform the relevant skill, so that the in-person session was able to further build on concepts and perfect skills rather than spend time focusing on basics. The 20-minute duration of the video prevented the wandering of attention that many students tend to experience in long sessions, and the playback speed feature was useful so students could watch it at different speeds as comfortable for them. Watching the videos also resulted in shorter in-person classes. Some students found the videos particularly helpful as they were specific to the paediatric population and were more personalized compared to videos generally available online.

Apart from improving the camera work and quality, videos should be better organized. This can be achieved with an objectives slide preceding each skill and the addition of a formal script and subtitles. Text boxes with examination checklists could also be added.

*“What helped was that we first had to see the videos and then come to the sessions. By seeing the videos, we already had a checklist, and an idea of how to perform and then performing under supervision really solidified all the concepts and all the steps” – (FGD_1_Participant 6)*

*“The videos were okay but not the best. They served as an overview before the session, but they could have been better content and execution-wise.”– (FDG_3_Participant 2)*

**Table 3:** Video quality and content, strengths, weaknesses, and suggestions for improvement

Video	Quotations
Quality	*“The videos need to be more zoomed in to the point that they zoom in and focus on the very area being examined – so you can then see the actual technique and learn.” – FGD_1_Participant 2* *“In some videos, the camera angle was not that good, and sound quality could also be improved.” – FGD_5_Participant 1*
Content	*“The video should match the actual in-person sessions. Because in the OSCE, I did something in the video and the facilitator pointed that it was wrong which was embarrassing because the faculty has told us to watch these videos.”* *– FGD_4_Participant 4*
Strengths	*“What helped was that we first had to see the videos and then come to the sessions. By seeing the videos, we already had a checklist, and an idea of how to perform and then performing under supervision really solidified all the concepts and all the steps” – FGD_1_Participant 6* *“Because a lot of us cannot watch long videos, so the short 20-minute videos helped us to not zone out.”– FDG_1_Particpant 1* *“The video was good because I could watch it at x1.5 speed to revise which was time-efficient, and we came into the session having some baseline knowledge.” – FGD_5_Participant 4*
Weaknesses	*“One or two of the videos had a mistake– in which what we were taught in the session was different from the video. There needs to be a disclaimer to point out the wrong stuff if the videos cannot be remade.” – FGD_1_Participant 3* *“The standard of videos - a lot of students use geeky medics, our videos were good, but I would still rather prefer to watch geeky Medics over the AKU videos simply because like they would say what we are examining and for what reason, with pictures sometimes of positive findings and that’s a more comprehensive experience in about the same time or even less time.” – FGD_2_Pariticpant 4* *“Some of the video formats are informal. They are also not very easily accessible before the exams for review even though we were able to find and open them easily before the session. And VLE is a software that doesn’t work that well, so there is a lot of inertia associated with it.” – FGD_5_Participant 7*
Improvements	*“Quality of the video, add pathophysiology merged with the real time examinations happening at that time, and fixing and focusing on angle.”* *– FGD_2_Participant 1* *“The videos need to perform examinations within 5–6 min instead of 20. Because then we also don’t learn how to do examinations within that time.”* *– FGD_3_Participant 5* *“As an institution, we do have the facilities to make the audiovisual a lot better than it is – and it is a good financial investment too because the current videos looked like they were made in 2008. Also, the videos were very casual, so it would be better to add a script, and a slide in the beginning with the objectives” – FGD_5_Participant 5*

*Acronyms: SP – Simulated Patient; VLE – Virtual learning environment, software; OSCE – Objective Structured Clinical Examination*

### Student experience

The intervention was well received by students across all five groups. Most found it to be an enjoyable learning experience, the activities were goal-directed and student-centered. Active participation and hands-on learning was important component of the intervention which was appreciated by all. Some students learned how to properly take consent, while others learned the correct technique of examining the general appearance of a patient; these were basic skills that were taught in previous years, however, had not been given the importance they required. At some points, the sessions became intensive and did result in saturation, however, overall, the environment was conducive to learning. Students were able to learn more about examinations of the various systems than they did in the related clinical rotations. Although the videos were too brief for some and required significant improvement, the following in-person sessions covered all the detailed information. The opportunity to give feedback at the end of the session was specifically appreciated by the students.

*“I think it was great out of all of the sessions we have had over the four years, this was one when I probably paid the most attention and learned the most out of this, even though technically we had learned most of this stuff beforehand” – (FGD_2_Participant 1)*

**Fig. 4:**
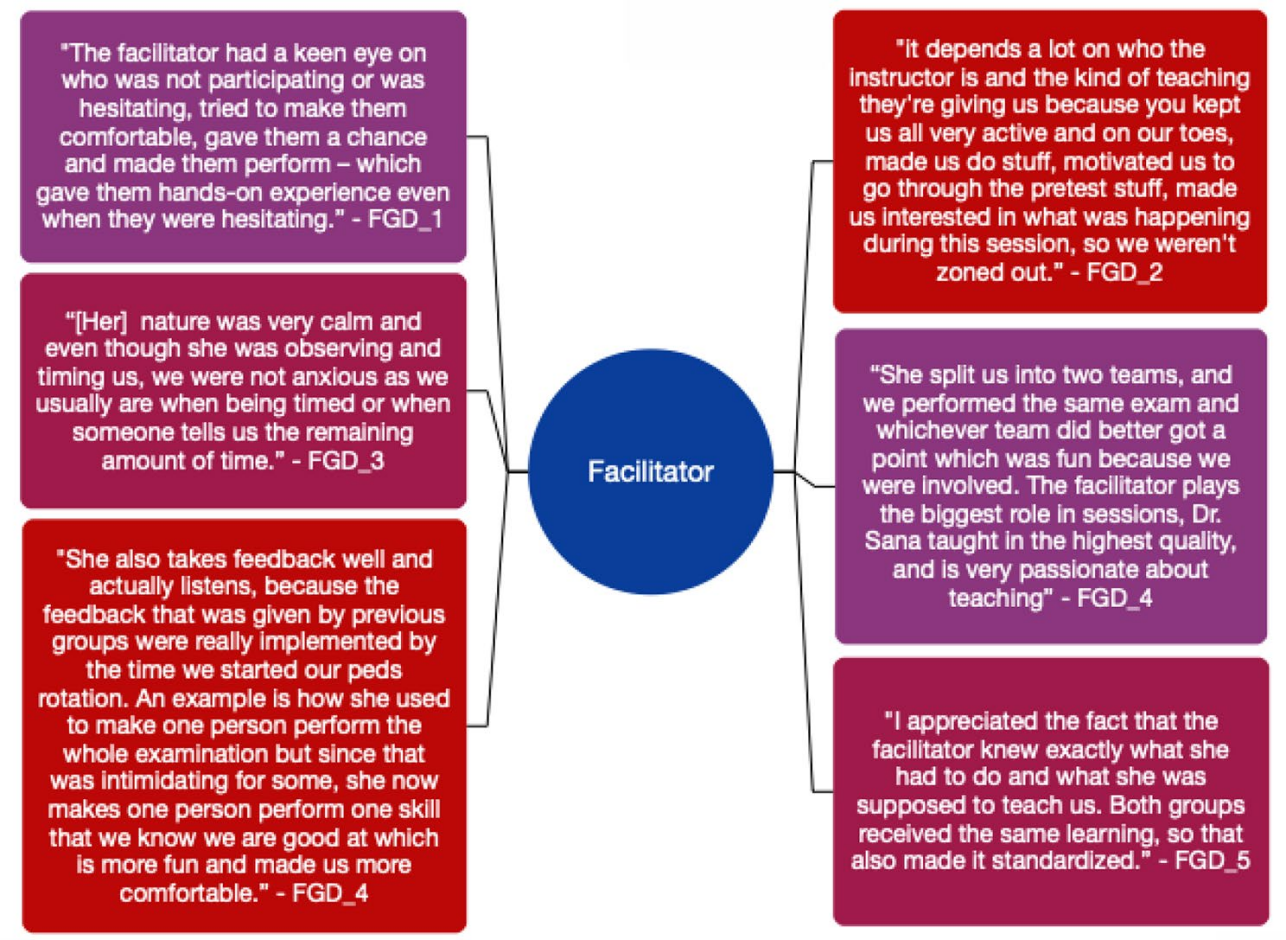
Student experiences with facilitator

The most significant importance was given to the facilitator conducting the in-person session. The interactive and innovative method of teaching, enthusiasm, and consistency in sessions, and the friendly and comforting environment created by the facilitator was the most lauded factor in the entire intervention. After completing the examination, the facilitator would also discuss clinical correlations which helped integrate clinical skills and pathophysiology. Figure 4 outlines the experiences of students from all five groups.

The intervention had an undeniably positive impact on students’ preparation for and performance in the clinical exam. In-person sessions were identified to be the most helpful and gave every student a significant level of comfort going into the exam. Most students felt that the examination part of their OSCE went well and was better than previous clinical exam experiences.

*“Revision was very easy and quick. Normally, we have to start studying from scratch, then memorize it but this time it was easier. And we were much more comfortable performing in the OSCE.” – (FGD_1_Pariticipant 1)*

*“This was the first time my examinations were better than my histories because of this [intervention] structure.” – (FGD_1_Participant 2)*

*“When I went to the OSCE, I didn’t revise my examinations very well, but I knew everything that I have to do. It was repeated so much that it was drilled into my head.” – (FGD_2_Participant 6)*

### Suggestions

The most frequent suggestion was having one examination per session, especially so for lengthy examinations like the cardiovascular and central nervous systems. Almost all the students found themselves losing concentration in sessions where two examinations were merged and taught together. Every student should have the chance to perform the activity, ideally on an SP. Learning should be assessed after the in-person session as well. Concluding the overall experience with an OSCE-like scenario involving a real patient, where the student undertakes history, examination, and discusses diagnosis and management with a supervisor would help to solidify all the concepts.

Specifically for cardiology, a bedside session involving a patient with positive findings would be helpful. Academic and clinical teaching should be integrated, as clinical faculty are not attuned to the way examinations are taught during academic sessions. The examination skills learned during the intervention should be practiced in clinics on real patients, with a facilitator observing students’ performance since real-life patient interaction is very different compared to learning on a simulator and requires additional skills.

The videos and quiz need significant work. Videos should be made according to checklists used by facilitators in OSCEs, and the quiz should be more interactive and targeted. Questions should be based on clinical scenarios and linked to the information shown in the preceding videos. Some students vouched for pre-recorded sessions which would be extensive, comprehensive easily accessible during a related rotation and before exams. While the facilitator of the sessions was praised by all, it is important to note that in future all the facilitators should be trained to teach the same way so that the sessions are standardized, and as effective as they were in this intervention.

Students should know the criteria according to which they are graded by consultants and should receive regular feedback. Many students felt that despite the in-depth sessions and detailed learning, there were still some steps that they supposedly missed during the OSCE. Being provided with the checklist and marking scheme that examiners have would help students identify what they missed out on and if they were taught that specific skill, so that it may be integrated in future sessions.

## Discussion

This study demonstrated that the hybridization of video-based learning with simulation significantly improved self-efficacy scores for all 4 examinations, and OSCE scores in the CNS and Abdomen groups. This is akin to a similar study by Jawaid et al. that reported higher OSCE scores following the introduction of blended learning, compared to traditional education (p = 0.49), [20] and a study carried out by Kim et al. where e-learning resulted in scores above the national average across all competencies tested [21]. The overall structure of the intervention was appreciated by all the students, who stated it allowed reinforcement of basic concepts, retention, and further insight into clinical applications. Likewise, a meta-analysis conducted by Hew et al. revealed medical students perceived video-based learning followed by an interactive discussion conducive to better comprehension, interest, and preparedness for the topic [12]. The sessions provided students an opportunity to learn from practice and feedback, as well as distinguish normal from pathologic findings, both of which served to increase students’ confidence. Therefore, systematic and scenario-based approaches can be used to increase competence without the fear of errors, as previously highlighted by Hecimovich et al. [22] Furthermore, the facilitator played the most significant role in the success of the intervention by interactive teaching, creating a student-friendly environment, and encouraging active learning, and is crucial to learner engagement and teamwork [23].

The response to the introduction of video-based learning specifically was mixed. Positive attributes identified by students included reinforcement of baseline knowledge, after which students were able to focus on applying concepts to the bedside during the session, short duration of videos, and flexibility of learning. These points have been included in recommendations by previous studies for optimizing video-based learning for medical education [24, 25]. However, students expressed reservations regarding the quality, camera work, and technical faults in the videos. Videos can be enhanced by using multiple camera angles, for example, the patient’s foot and right ends, including examination checklists used to grade students in the OSCE, and adding audiovisual features of the mentioned pathologies. Videos should also be optimized for the platform they are uploaded to and tested before implementation. In case of limited resources, sharing resources has been identified as an efficient way to support learning. Additionally, as students may face challenges in video-based learning without interacting with others, future research on integrating interactive tools in this setting is relevant [26].

Although post-video quizzes were appreciated as an initiative, they did not represent the content covered in the video, nor was an answer key provided upon completion. Quizzes can be improved by incorporating real-life scenarios, testing pathophysiology and management, and including detailed explanations; learning objectives should be the same as for the video. This approach has been indicated to bridge the gap between simulated teaching and clinical application [27]. Moreover, students perceived simulators to be effective (particularly for cardiovascular and respiratory examinations) but did encounter limitations. As simulators are a highly effective tool in medical education, [28, 29] they should be refined, for example, accounting for differences in adult and paediatric findings, and personalizing mannequins used in examinations.

This study had a few limitations. First, the data has been collected for a single institution and may not be representative of the entire population. Secondly, the impact of the intervention on student performance was studied using correlation analysis only. A multivariate analysis may enable a better understanding of the multiple factors that influence student performance.

## Conclusion

Hybridization of video-based learning with simulation-based teaching for flipping the clinical skills teaching of medical students facilitated in providing an optimum opportunity for deliberate practice, in-depth reflection, tactful problem solving, and clinical reasoning. This study provided significant evidence about the role of hybrid model of teaching on the acquisition of clinical and analytical skills of medical students in a resource limiting setting of a low middle income country. Along with this the student experience of learning through this model will facilitate the course developer, medical educationist, and clinical teachers to enhance the clinical teaching and learning in relevant disciplines. It will be worth noticing the longitudinal impact of this intervention on the subsequent performances of graduate students and ultimately on patient care in future studies.

## Data Availability

All data generated or analyzed during this study are included in this published article.
